# A Method for Investigating Access to Diaper Changing Stations in Restaurants

**DOI:** 10.7759/cureus.18810

**Published:** 2021-10-15

**Authors:** Nishant Pandya, Rachel Granberg, Russell K McIntire

**Affiliations:** 1 College of Population Health, Thomas Jefferson University, Philadelphia, USA

**Keywords:** diaper changing stations, children, restaurants, restrooms, phone interviews

## Abstract

Parents and caretakers of young children need diaper changing stations (DCSs) to fully utilize public and commercial spaces, but few studies measure their availability. We developed a method for assessing the availability of DCSs in restaurant restrooms through phone interviews and validated the results using in-person visits. This study tested a phone interview methodology for investigating availability within restaurants, and the extent to which DCSs were accessible to both male and female caregivers. In May of 2019, phone interviews were conducted to survey the employees of 60 Philadelphia restaurants with a public restroom available to patrons to determine whether they had unisex or gendered restrooms, a DCS, and accessibility to a DCS for both male and female caregivers. Each interview was followed by an in-person audit. During phone interviews, 10 (16.7%) restaurants reported having and 50 (83.3%) reported not having DCSs. In-person audits confirmed 59 of 60 (98.3%) phone interview responses about the presence of DCSs (Cohen’s kappa: 0.94) and 55 of 60 (91.7%) reports of restroom types (Cohen’s kappa: 0.83). In this study, the phone interview methodology accurately identified the presence of DCSs in restaurants. This methodology can be used to identify disparities and to advocate for policy changes to improve access to DCSs for all who need them.

## Introduction

While parents can curate their home environments to fit their family’s needs, spaces outside the home are not always designed with young children in mind. In commercial spaces such as restaurants, the institutional design reflects values by demonstrating which groups are provided access and which groups are marginalized through failure to meet specific needs [1]. This principle applies when considering accessibility for parents with young children. Amenities often offered for families in restaurants include storage for strollers or highchairs for patrons with children. While storage for strollers and highchairs, when available, is accessible for any caregiver, diaper changing stations (DCSs) access is relegated to bathrooms, which may limit availability to certain caregivers based on gendered stalls.

Since the late 1900s, there has been a change in parenting roles characterized by increasing paternal engagement. No-fault divorce laws and the increasing proportion of dual-income households facilitated the rise of the working mother population between 1970 and 1990 [2]. Furthermore, DCSs need to be accessible to both mothers and fathers, as men have become more active in childrearing compared to rates in the past [3]. Fathers now comprise 17% of all stay-at-home parents in 2016, compared to 10% in 1989, indicating an increasing involvement in childrearing activities [4].

As parenting responsibilities have become more equitable between genders, there has been a push to increase male caregiver access to parenting resources in public spaces, notably DCSs. The Bathroom Accessible in Every Situation (BABIES) Act [5] required all federal buildings to make DCSs accessible in male and female restrooms. While the law was implemented nationally, it was limited to only federal buildings; however, a groundswell of activity has been building upon the BABIES Act to establish new DCS regulations. The states of California, Illinois, New York, and Maryland, and multiple US cities have passed legislation that requires all new public buildings or buildings undergoing large renovations to install DCSs in a manner accessible to both men and women [6-8]. Alabama, Massachusetts, Minnesota, New Hampshire, New Jersey, New Mexico, Tennessee, Washington, and Wisconsin have active bills within their respective state house or senate as of February 2020, that would require new businesses or public buildings to install DCSs [9-13]. In most major cities, no laws exist requiring DCS installation outside of federal buildings that are funded through tax revenue, as outlined in the BABIES Act [14]. Some non-government platforms have attempted to collect and report locations of DCSs for parents, though these data are neither complete nor systematic.

Despite the growing body of legislation on DCS access, there are only limited studies that have attempted to study the local availability of DCSs. Between 1998 and 2005, researchers studied the number of DCSs installed in 200 locations in Albuquerque, New Mexico. Phone calls were used to gather the data in 1998, and the research team visited the locations in 2000, 2003, and 2005 to measure the change in access to DCSs for men. This study utilized both phone interviews and physical visits, but the initial data collection was not verified until the first visit two years later [15]. Additionally, the Chancellor’s Committee on the Status of Women at the University of Illinois Urbana-Champaign led the efforts through her department to collect data on all 1,875 restrooms on campus to note access to DCSs and lactation rooms by physically visiting each location [16].

Conducting in-person visits to physically evaluate locations is a good way to assess what resources are available. This methodology avoids self-reporting by another party and ensures accurate data collection. However, the methodology of visiting locations is time-consuming, resource-intensive, and may limit the research to a smaller geographic area. The limitations of this methodology may explain the scarcity of research on this resource, inhibit advocacy efforts, and ultimately, prevent comprehensive access to DCSs in restaurants.

Thus, although the methodology of phone interviews to collect data on DCSs has been employed in the past, our study aimed to validate the accuracy of phone interviews with timely subsequent audits. The purpose of this study was to assess the reliability of a methodology to determine whether restaurants had DCSs. We hypothesized that phone interviews would accurately identify the presence of DCSs in restaurant bathrooms.

## Materials and methods

Phone interviews were conducted among employees of Philadelphia restaurants to assess whether or not restrooms had DCSs installed. Additionally, interviews identified restroom types and DCS accessibility. The subsequent audits assessed the accuracy of the phone interview for each restaurant. The study was approved by the Thomas Jefferson University Institutional Review Board (19E.055).

Sample selection

The study interviewed respondents from 60 randomly selected restaurants in the Philadelphia zip code 19103. Zip code 19103 was chosen out of convenience, based on its geographical proximity to researchers at Thomas Jefferson University and high restaurant density. A list of restaurants was obtained in November 2018 via the Philadelphia Department of Public Health Food Safety Inspection Database [17]. The inclusion criteria used to generate the list of all restaurants included zip code (19103), facility type (restaurant), and facility sub-type (eat-in). These criteria removed take-out locations, food trucks, cafeterias, corner stores, and other food establishments that sell food but are not designed primarily for dine-in meals.

**Figure 1:**
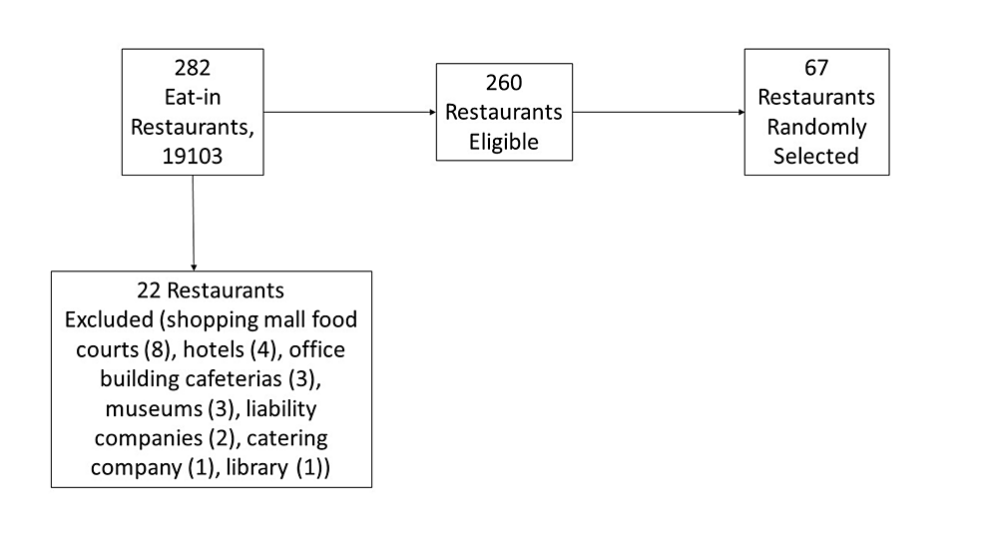
Sample selection flow diagram.

This total list of restaurants included 282 results, of which 22 (7.8%) were excluded by the researchers who determined that these institutions were not primarily free-standing restaurants at which patrons would eat. The 22 omissions included eight restaurants in a shopping mall food court, four in hotels, three cafeterias inside office buildings, three in museums, two in rental limited liability companies, one catering company, and one public library. The remaining 260 restaurants were sorted in alphabetical order by restaurant name. Using an online random number generator, 67 restaurants were selected for data collection (see Figure 1).

Data collection

The primary researcher conducted phone interviews using an IRB-approved instrument (Appendix A). Respondents from five restaurants declined to participate. Participating respondents were asked whether their restaurant had unisex single stalls, gendered restrooms, or both. Only two restaurants were excluded because they only had restrooms for staff. If a restroom was available for patrons, respondents were asked whether it had a DCS. If the restaurant had unisex restrooms, it was assumed the DCS would be accessible by both men and women. If the restaurant had gendered restrooms or both types, respondents were asked whether the DCS was installed in both the men’s and women’s rooms.

In-person audits were completed at each of the remaining 60 restaurants within one week of the phone call to assess the validity of the responses from the phone interviews and assess the presence of a wall-mounted DCS installed for the sole purpose of changing children’s diapers. If an employee approached the auditor, a scripted request describing the study was shared to gain access and assess the restroom type and presence of DCS. In eight instances, the restaurant’s restrooms were only available to patrons. In those instances, the auditor purchased a small item to become a customer and complete the audit, using private funds from the principal investigator. Restroom types and installed DCSs were independently assessed by auditors.

Data analysis

The primary goal was to evaluate the accuracy between phone interview and in-person audit results. Descriptive statistics were calculated regarding restroom types and prevalence of restaurants with restrooms containing DCSs among restaurants in the sample. Percent agreement between the phone calls and in-person audits was calculated using Cohen's kappa and evaluated using a statistical significance level of P ≤ 0.05.

## Results

Of the 67 restaurants called by researchers, two restaurants were ineligible for inclusion as they did not have restrooms for customers, and five respondents declined to participate, resulting in a participation rate of 92.3% (60/65).

Phone interviews reported 16.7% (10/60) of restaurants had DCSs (Table 1). Of the 10 reporting DCSs, five reported having them in unisex stalls, three in gendered stalls, and two reported them in both unisex and gendered stalls. The two restaurants with both unisex and gendered stalls reported having DCSs in only one unisex stall. Only one of the three restaurants with only gendered restrooms reported having DCSs in both men’s and women’s restrooms. The other two restaurants reported having DCSs only in the women’s restroom. Respondents correctly self-reported their restaurant’s DCS status 98.3% (59/60) of the time, with a strong agreement between the two methods of data collection (Cohen’s kappa: 0.94). The only discrepancy occurred in one restaurant that reported having a DCS in a unisex stall, which was not present during the in-person audit. All 50 restaurants that reported not having a DCS did so correctly.

**Table 1:** Bathroom types and diaper changing station presence in 60 Philadelphia, Pennsylvania restaurants in May 2019 using phone interviews and follow-up in-person audits. DCS, diaper changing stations.

Results from phone interview			Results from in-person audit		
Bathroom type	Number of restaurants (N = 60)	Number of restaurants with DCS	Bathroom type	Number of restaurants (N = 60)	Number of restaurants with DCS
Unisex stalls	40	5	Unisex stalls	39	4
Gendered stalls	16	3	Gendered stalls	17	3
Unisex stalls and gendered stalls	4	2	Unisex stalls and gendered stalls	4	2

Respondents correctly reported their restaurant’s type of restroom 91.7% of the time (55/60), with a strong agreement between the two methods of data collection (Cohen's kappa: 0.82). Unisex restrooms were reported correctly 92.5% (37/40) of the time with three gendered restrooms misreported as unisex. Gendered restrooms were reported correctly 87.5% (14/16) of the time with two unisex restrooms misreported as gendered. Restrooms that had both unisex and gendered stalls reported correctly 100% (4/4) of the time.

Of the 60 restaurants included in the study, four (6.7%) had a restroom accessible to its patrons but disclosed that the restroom was owned by the building from which the restaurant leased space; thus, the decision to install DCSs was made by the building owner. All four restaurants in this category were unisex stalls with no DCSs and reported so correctly.

## Discussion

The objective of this study was to assess a methodology of using phone interviews followed by in-person audits to determine whether restaurants had DCSs available for caregivers. In this study, the phone interview methodology accurately identified whether restaurants had DCSs 98.3% (59/60) of the time (Cohen’s kappa: 0.94), suggesting that phone interviews may be a valid methodology for determining the presence and accessibility of DCSs. The phone interview also accurately collected data on the type of restroom at the restaurant 91.7% (55/60) of the time (Cohen’s kappa: 0.83). Based on this study, we found the accuracy of the phone interview methodology to gather this information to be valid. Restaurant employees were the likely respondents to the phone interviews. These employees also are likely to use and possibly clean the restaurant restrooms, making them credible sources of knowledge about the referenced restroom. Additionally, in this study, not all respondents knew the answers to the phone interview questions, but they were able to obtain an answer by asking another employee or by checking the restroom to confirm their responses.

The few research studies published on this topic report using the resources-intensive process of physically evaluating all restrooms or fail to verify the results of telephone surveys with in-person audits [15,16,18]. The methodology in the current study suggests that future studies could conduct phone interviews to accurately identify the presence of DCSs in restaurants, without using the time- and resource-intensive process of physically visiting locations. This work is critical to increasing the body of research on DCS accessibility in public spaces and identifying demographic and geographic disparities in access. This methodology revealed only 15.0% (9/60) of restaurants had DCSs installed. While based on a limited sample size in a small geographic area, the results do support concerns about inadequate DCS availability communicated by advocates. While advocates have not demanded action to quantify DCS access, they repeatedly report the need is not currently being met, as evidenced by the attention DCS access has received through local news outlets. Quantifying the number of DCSs is a necessary step, as understanding the extent to which DCSs are available can direct advocacy efforts to build more facilities with DCS access.

The methodology also revealed access to DCS may be greater for women than men. While 15% (9/60) of restaurants had DCSs installed, only 11.7% (7/60) were accessible to men, as two restaurants had DCSs installed only in the women’s restroom. This further supports the advocacy claims that DCS access for fathers is more limited. Only one other study has explored differences in access to DCS by gender and results similarly showed less access for men [15].

There were seven instances where respondents to the phone interview volunteered that there was a countertop, table, or another flat platform large enough to change a diaper within the restaurant bathroom. While auditors were able to verify that these spaces were present, they were not considered DCSs in this study due to important sanitary and safety concerns. Dirty diapers are a source of potential infectious disease, and the contamination of spaces with fomites presents a considerable disease risk. One study identified that changing diapers was positively associated with giardiasis infections for those without a travel history, while another showed that contaminated DCSs themselves can be a nidus for a norovirus outbreak [19-20]. Changing diapers in an area not indicated for this use may result in inadequate cleaning measures by restaurant staff and an increased risk of infection among unknowing patrons who then utilize these surfaces. Beyond infection prevention, there are additional child safety concerns, as raised platforms and countertops without the traditional safety features offered by standard DCSs, such as straps and concaved beds, may present a risk to a child rolling or falling.

Limitations of this study include a small sample size of only 60 restaurants and a limited geographic span. Because the geographic distribution is limited, we cannot make any claims that the sample of 60 restaurants is representative of a larger population. Although the sample and study design demonstrated that phone interviews are a valid tool for identifying the presence of DCSs in restaurants, the study does not allow for broader conclusions to be reached regarding significant differences in access to DCSs between fathers and mothers. Of note, three eligible subjects hesitated to participate due to fear of penalty for not having a DCS, despite the purpose of this study being clearly stated. Improving the introductory message to emphasize its academic nature and absences of regulatory goals may help assuage these concerns.

Future directions for this research include using this methodology to create a larger database of DCS installation in restaurants. This information can be shared with local governments for record-keeping or creating resource guides for parents. Research can also study DCS access by zip code and income, self-advertising “kid-friendly” restaurants, chain restaurants, and independent restaurants. It can reveal disparities or compare accessibility among restaurants when viewed by different demographics, such as gender or restaurant characteristics. Quantifying access to DCSs may also serve to bolster advocacy efforts to create policies requiring their installation in new businesses.

## Conclusions

Researchers found strong agreement between phone interviews and audits when collecting data on the presence of DCSs and the type of restrooms among Philadelphia restaurants. Phone interviews can be used to continue collecting data to generate more complete databases of the presence of DCSs in restaurants. The information resulting from the future use of this methodology can serve as a resource for parents and caretakers, as record-keeping for local governments, or to stimulate future advocacy work for parental access to DCSs.

## Appendices

Survey questions

"Hello, my name is NAME and I am a researcher at INSTITUTIONAL AFFILIATION. I am completing a study about access to diaper changing stations in restaurants in Philadelphia and wish to ask a few questions about the presence of diaper changing stations in your location. I also have two questions for the owner of the restaurant should they be available. Would you be willing to answer a few questions? This would take approximately two to three minutes and require no private information."

"What type of bathrooms do you have installed at your restaurant, single-stall or gendered bathrooms?"

(If no bathroom, restaurant meets exclusion criterion)

(If yes) "Does your restaurant have a diaper changing station installed?"

(If the answer to the previous question was gendered bathrooms) "Is the diaper changing station(s) in the men’s room(s)? women’s room(s) or both?"
